# Supplementary material for: High Fat High Sucrose Diet Modifies Uterine Contractility and Cervical Resistance in Pregnant Rats: The Roles of Sex Hormones, Adipokines and Cytokines
Source: Life (Basel). 2022 May 26;12(6):794. doi: 10.3390/life12060794 (PMC9224847; doi:10.3390/life12060794)
Supplement: Supplementary file 1 [file life-12-00794-s001.zip › life-1733076-supplementary.pdf]

Figure S1. Full gel photos of adipokines Western blot measurements.

KissR

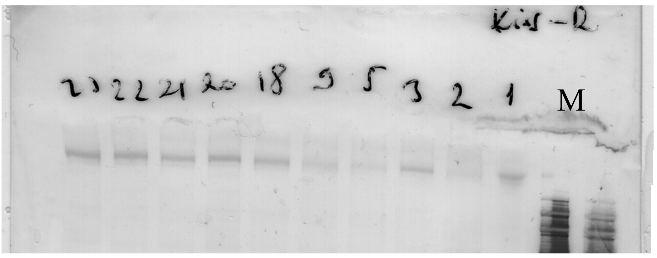

57 kDa
42 kDa
31 kDa

Actin

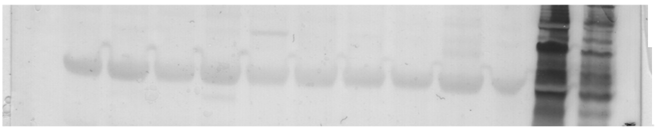

72 kDa
57 kDa
42 kDa

Lanes 1, 2, 3, 5, 9: the numbers of standard diet rats
Lanes 18, 20, 21, 22, 23: the numbers of standard diet rats
M: molecular weight markers

Ob-R/Leptin receptor:

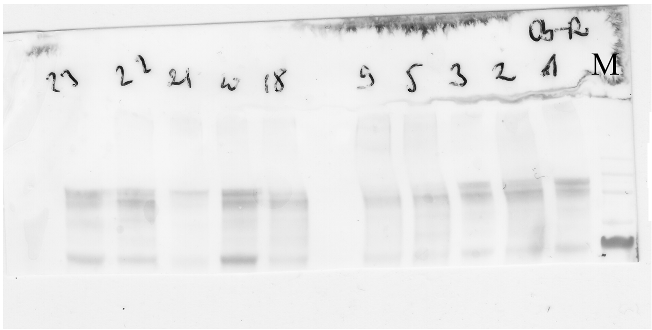

240 kDa
165 kDa
125 kDa
93 kDa

Actin

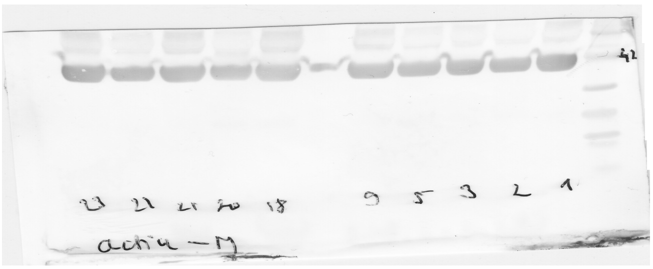

57 kDa
42 kDa
31 kDa
24 kDa
18 kDa

Lanes 1, 2, 3, 5, 9: numbers of standard diet rats
Lanes 18, 20, 21, 22 ,23: numbers of standard diet rats
M: molecular weight markers

Adiponectin 1 receptor

23 22 21 20 19 18 9 5 3 2 1 M

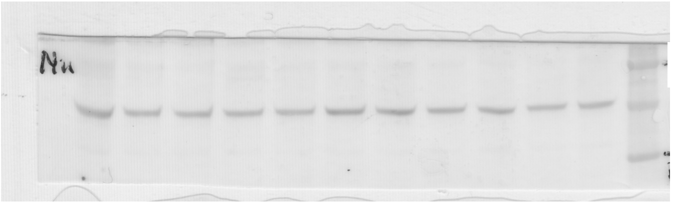

57 kDa
42 kDa
31 kDa

Actin

23 22 21 20 19 18 9 5 3 2 1 M

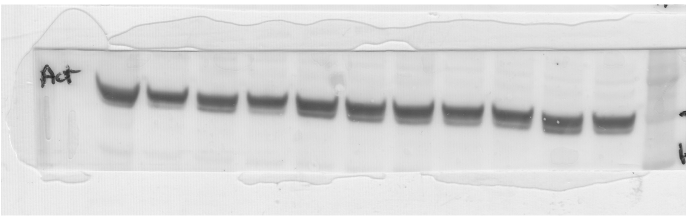

57 kDa
42 kDa
31 kDa

Lanes 1, 2, 3, 5, 9: the number of standard diet rats
Lanes 18, 19, 20, 21, 22, 23: the number of standard diet rats
M: molecular weight markers

Adiponectin 2 receptor

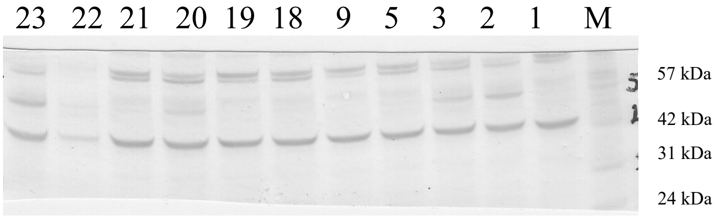

Actin

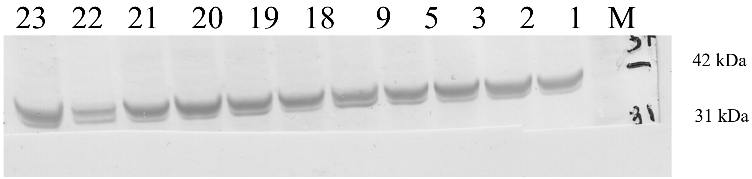

Lane 1, 2, 3, 5, 9: the number of standard diet rats
Lane 18, 19, 20, 21, 22, 23: the number of standard diet rats
M: molecular weight markers
